# 

**Published:** 1821-01

**Authors:** 


### THE LONDON MEDICAL AND PHYSICAL JOURNAL.

CONTAINING

ORIGINAL CORRESPONDENCE OF EMINENT PRACTITIONERS,

AND CRITICAL ANALYSES OF NEW WORKS

RELATING TO

*MEDICINE, SURGERY, CHEMISTRY, PHARMACY, BOTANY, AND NATURAL HISTORY.*

---

*Successively conducted by*

T. BRADLEY, M.D.

A. F. M. WILLICH, M.D.
R. BATTY, M.D.
A. A. NOEHDEN, M.D.
J. ARNEMAN, M.D.
J. P. PFAFF, M.D.
J. ADAMS, M.D.
W. SHEARMAN, M.D.
W. ROYSTON, Esq.

SAMUEL FOTHERGILL, M.D.

AND

WILLIAM HUTCHINSON, M.D.

MEMBER OF THE SOCIETY OF THE COLLEGE OF PHYSICIANS OF PARIS
FELLOW OF THE LINNEAN SOCIETY,
AND OF THE PHYSICAL AND MEDICAL SOCIETY OF BONN
ON THE LOWER RHINE;
MEMBER OF THE MEDICAL AND CHIRURGICAL SOCIETY OF LONDON;
AND ONE OF THE PHYSICIANS TO THE ROYAL METROPOLITAN
INFIRMARY FOR SICK CHILDREN.

---

VOL. XLV.

FROM JANUARY TO JUNE, 1821

Et quoniam variant morbi, variabimus artes;
Mille mali species, mille salutis erunt.

4627

---

*LONDON:*

PRINTED FOR THE PROPRIETORS,

BY J. AND C. ADLARD, 23, BARTHOLOMEW CLOSE;

PUBLISHED BY J. SOUTER, 73, ST. PAUL'S CHURCH-YARD; AND

MAY BE HAD OF ALL BOOKSELLERS.

W1
LO 31

### ANALYTICAL TABLE OF THE CONTENTS

OF

THE FORTY-FIFTH VOLUME

OF THE

### London Medical and Physical Journal.

### DESCRIPTIVE ANATOMY.

Page.

Of the Villosities of the Intestines . . . *Proëmium* iii.

Of some points in the development of the Vertebræ . . — iv.

Lymphatic System . . . — v.

structure of the Teeth . . . — vi.

Of a new-discovered Nerve in the Ear . . . . — v.

Of the dermoid texture of the Negro . . . . . 83

Of the thyroid gland of the Horse . . . . . . . 284

Of a structure in the nostrils of the Horse . . . . . 321

Of the Absorbent Vessels . . . . . . . . 411

Of the udder of the Cow . . . . . . . . 462

### PHYSIOLOGY.

Vital Phenomena, *in general.*
Reflections on the nature of vitality . . . 238, 441
Views of the general relations of the vital phenomena in different seats . . . . . . 241, 245, 342
Effects of climate and certain modes of civilization on the character of the . . . . . . . . 341, 429
Nervous and Sensitive Systems, *in general.*
On different modes of sensibility . . . . . . 234
Phenomena of excitement of the brain . . *Proëm.* xxxi.
Nutritive Functions (*organic*, of Bichat).
On the means of nutrition, in general . . . . . 340
On the properties and functions of the mucous membranes 245, 427
*Appetite for, and administration of, food.* Origin of . . . 242
*Mastication.* Influence of . . . . . . 369, 370
*Insalivation* . . . . . . . .
*Deglutition* . . . . . . . . .
*Digestion.* The origin and phenomena of rumination in man, *Proëm.* xxvii. 362
*Absorption.* Various considerations on the agents and phenomena of, 226, 233, 411, 435
*Circulation.* Mechanism of, in general . . . . *Proëm.* ix.
Influence of respiration on the . . . . . — xvi.
Phenomena of, dependant on different relative quantities of blood 228
*Respiration.* Some points in the mechanism of, especially on the synergy of the glottis and abdominal muscles, and the functions of the diaphragm . . . . . . *Proëm.* xviii.
*Assimilation.* - Formation of bone . . . *Proëm.* xxviii. lvii.
*Secretion.* Properties and qualities of human urine . . . . 385
Urine of some species of Rana . . . . . . 251
*Excretion* . . . . . . . . .

Page.

CALORIFIC FUNCTION.
ANIMAL FUNCTIONS, *in general.*
Phenomena attendant on muscular exertions . *Proëm.* xviii.
*Sight.* Considerations on some points in the theory of vision *Proëm.* xxiii.
*Hearing.* Considerations on some phenomena of; especially on its connexion with the nerves of touch in general . . . 318
*Smell* . . . . . . . . .
*Taste* . . . . . . . . .
*Touch.* Influence of the organs of, on the sense of hearing . . 318
PERCEPTIONS (*Intellectual*), AND THEIR RESULTS.
Results of different modes and degrees of sensibility and sensation 234
Origin of the instincts and passions . . . . 242, 250
*Movements.* On the mechanism of muscular efforts . *Proëm.* xviii.
*Sleep* . . . . . . . . .
*Voice* . . . . . . . . .
GENERATIVE FUNCTIONS.
*Proper effects of.* Hybrid of the mare and quagga, produced under extraordinary circumstances . . . . . 172
*Preparatory to the generative act* . . . . .
*Gestation* . . . . . . . .
*Parturition* . . . . . . . .
*Lactation* . . . . . . . .
*Sexual characteristics* . . . . . .
PECULIARITIES IN THE FUNCTIONS AT THE DIFFERENT PERIODS OF LIFE.
*Fœtal life* . . . . . . . .
*Infancy* . . . . . . . . .
*Childhood* . . . . . . . .
*Puberty.* Precocious development of . . . . . 345
*Manhood* . . . . . . . .
*Old age* . . . . . . . .
PECULIARITIES IN THE FUNCTIONS OF INDIVIDUALS.
Temperaments and Idiosyncracies, *in general* . . . 240
*in particular* . . . 244

### PATHOLOGY.

GENERAL REMARKS.

Treatise on the Elements of . . . . *Proëm.* xxix. 225

On the origin of disease, in general . . . . . 336

OBSERVATIONS RELATIVE TO.

ABSCESS, *urinary* . . . . . . . 166

AMAUROSIS . . . . . . . 252

ANGINA PECTORIS . . . . . *Proëm.* lvi.

ASTHMA . . . . . . . . 250

BONES, softening of the . . . . *Proëm.* lxx.

fractures of the, re-union of . . . *Proëm.* lxxvi.

BRAIN, pultaceous disorganization of the . . . *Proëm.* lii.

Erethism and inflammation of the . . . . . 476

Concussion of the . . . . . . . 208

Hernia of the . . . . . . . . 195

BRONCHITIS . . . . . . . . 374

CALCULOUS DISEASES . . . . . . . ib.

COUGH, from disease of the liver . . . . . . 492

COW-POX . . . . . . . . 267, 277

CROUP . . . . . . . . . 374

EMPHYSEMA . . . . . . . . 476

EYE, diseases of, in general . . . . *Proëm.* lxi. 135

FEVER, *in general* . . . . . *Proëm.* xxxii.

*puerperal* . . . . . *Proëm.* lii. 122

*yellow* . . . . . . *Proëm.* xxxvii.

FRACTURES, of the bones . . . . *Proëm.* lvii. lxx. lxxv.

| | Page. |
|---|---|
| GANGRENE, of the face | 492 |
| HEMORRHAGE, from the spleen | 473 |
| HERNIA CEREBRI | 195 |
| HYDROPHOBIA | 280 |
| INFLAMMATION, phenomena of, in general | 429, 430, 470 |
| Its connexion with fever | *Proëm.* xlvii. |
| Varieties of | 241 |
| of nerves | 323 |
| INTESTINES, expulsion of a portion of the, by the anus | *Proëm.* lxix. |
| IRRITATION, general effects of | 428 |
| MUCOUS MEMBRANES | 245, 250 |
| NERVES | 315 |
| OPHTHALMIA | *Proëm.* lxi. |
| PLAGUE | *Proëm.* xlix. 135 |
| SMALL-POX | 475 |
| SPINAL DISEASES | *Proëm.* lvi. 103 |
| SPINAL MARROW | 282 |
| URETHRA, rupture of the | 166 |
| TUMORS | 488 |
| VENEREAL DISEASES | *Proëm.* lx. |
| VARIOLOID DISEASES | 475 |
| WORMS, *intestinal* | 71, 192 |
| WOUNDS, of nerves | 329 |

### PATHOLOGICAL ANATOMY.

| | |
|---|---|
| Of the subjects of fever | *Proëm.* lxxi. |
| Pultaceous disorganization of the brain | — lxxii. |
| Tumors of nerves | — lxxii. 326 |
| Of the subjects of asthma | — lxxiii. |
| Rupture of the heart | — lxxiii. |
| Fractured neck of the femur | — lxxv. |
| Inordinate number of the vertebræ | — ib. |
| Transposition of the thoracic and abdominal viscera | — lxxvi. |
| Duplication of several muscles | — ib. |
| Lacrymal calculi | — lxxvii. |
| Singular ossification in the spleen | 15 |
| Bicephalous fetus | 128 |
| Rare case of mal-formation of a fetus | 199 |
| Osseous tumor in the uterus | 353 |
| Remarkable case of psoas abscess | 458 |

### NOSOLOGY.

| | |
|---|---|
| Considerations on the principles of | 235 |

### SEMIOLOGY.

| | |
|---|---|
| RELATIVE TO GROUPS OF SYMPTOMS, OR DISEASES. | |
| Bronchitis and laryngitis | 374 |
| Inflammation and irritation | 241 |
| RELATIVE TO PARTICULAR ORGANS, STRUCTURES, AND FUNCTIONS. | |
| Those of the mucous membranes | *Proëm.* lxxvii. |
| Those of the lymphatic system in the plague | — 1. |
| The buffy coat on the blood | 85 |
| The pulse | 41 |

### HYGIENE.

*Page.*

Relative to contagious fever . . . . . *Proëm.* lxxx.

- the plague of Egypt . . *Proëm.* xlix. 35, 211, 287, 467

- vaccination . . . . . . 265, 277

- to poisons . . . . . . . 257

- the origin of urinary calculi . . . *Proëm.* lxxxi. 235

### THERAPEUTICS,

GENERAL CONSIDERATIONS ON.
- Observations on some general points of . . . 339, 520

OBSERVATIONS RELATIVE TO

- ABSORPTION, considered in its therapeutical relations . . 337

- BRAIN, various affections of, in infants . . . . 478

- CANCER . . . . . . . . 27, 89

- EYE, diseases of, in general . . . . . 135

- FEVER, the use of mercury in . . . *Proëm.* lxxxv. 492

  - *puerperal* . . . . . . . 122

- GONORRHŒA, the use of cubebs in . . . . 463

- FRACTURES of the bones . . . . 22, 356, 169

- HERNIA, *intestinal* . . . . . . 19

  - *cerebri* . . . . . . . 195

- HYDROPHOBIA . . . . . . . 255

- IDIOSYNCRACIES and TEMPERAMENTS . . . . 240

- INFLAMMATION, in general . . . . . 516

- INTESTINES, obstruction of the . . . . . 1

- MELÆNA . . . . . . . . 496

- NERVES, diseases of, in general . . . . . 315

- OPHTHALMIA . . . . . . . 137

- PARALYSIS, in general . . . . . . 302

- POISONS, *corrosive sublimate* . . . . . 498

- RHEUMATISM . . . . . . . 66

- ULCERS . . . . . . . . 32, 450

- URETHRA, rupture of the . . . . . 166

- WORMS, *intestinal* . . . . . . . 71

### CHIRURGERY.

*Respecting the Operations for*

- ANEURISM . . . . . . . . 254

- ARTERIES, arresting of the circulation in . . . 169

- ARTIFICIAL ANUS . . . . . . . . 1

- LOOSE CARTILAGES IN JOINTS . . . *Proëm.* lxxxv.

- STONE IN THE BLADDER . . . — ib.

- TRACHEOTOMY . . . . . . . 489

- TUMORS . . . . . . . . . 165

### OBSTETRICISM.

On the spontaneous evolution of the fetus . . *Proëm.* lxxxviii. 167

Case of rupture of the vagina . . . . . . . 496

Various observations respecting . . . . . . . 499

On rupture of the uterus . . . . . . . . 502

### MEDICAL JURISPRUDENCE AND MEDICAL POLICE.

On the doctrine of the contagion of the plague *Proëm.* l. 35, 211, 287, 467

On medical education and practice . . . . . 177

### CHEMISTRY.

| | Page. |
|---|---|
| Analysis of human bile containing cholesterine . . | *Proëm.* lxxxviii. |
| Sources of iodine . . . . . . . . | — lxxxix. |
| Analysis of asarum europæum . . . . . . | — ib. |
| serpentaria . . . . . . | — xc. |
| Sulphate of platinum a test for gelatine . . . . . | 174 |
| Tests for some mineral poisons present in certain coloured liquors . | 200 |

### MATERIA MEDICA, PHARMACY, MEDICAL BOTANY, AND SOME POINTS IN NATURAL HISTORY.

| | |
|---|---|
| Extract of the solanum tuberosum, recommended as a medicine | *Proëm.* xci. |
| The lepidium ruderale, recommended as a medicine . . | — ib. |
| Treatise on the Medicinal Use of the Prussic Acid . . . | 45 |
| Considerations on compression and percussion, as a remedy . . | 66 |
| Efficacy of cubebs in gonorrhœa . . . . . . | 467 |
| Treatise on the Medicinal Use of the Colchicum Autumnale . . | 218 |
| Acetate of lead, medicinal effects of . . . . . . | 252 |

### MISCELLANEA.

| | |
|---|---|
| Magnetic effects of electricity . . . . . . . | 83 |
| Comparative view of mean temperature at different places in Great Britain . . . . . . . . . . | 86 |
| Reports of Diseases for London . . . . . . | 87, 251 |
| Inoculation of the scab in sheep . . . . . . . | 173 |
| Account of some supposed instances of spontaneous combustion of the human body . . . . . . . . . | 347 |
| Biographical notice of the late Dr. Gregory . . . . | 437 |
| Meteorological Journals . . . . | 88, 175, 264, 352, 440, 528 |
| Prize Questions . . . . . . . . . . . | 349 |
| Monthly Catalogues of Books . . . . | 175, 263, 353, 400, 527 |

---

### NAMES OF AUTHORS,

*Of whose Works, Observations, &c. either a detailed Account, or more or less general View, is given in*

THE FORTY-FIFTH VOLUME OF THE

### London Medical and Physical Journal.

| | |
|---|---|
| Alard, Dr. of *Paris*, Considerations on the arrangement and functions of the Absorbent System in health and disease . . . | 411 |
| Balfour, Dr. W. of *Edinburgh*, Illustrations of the efficacy of Percussion and Compression in the cure of Rheumatism, &c. . . | 66 |
| Bampfield, Mr. of *London*, case of a large Encysted Ossification in the Spleen . . . . . . . . . . | 15 |
| Barde, Dr. of *Paris*, cases of Rupture of the Heart . | *Proëmium*, lxxiv. |
| Beclard, Dr. of *Paris*, on Ossification . . . . . | *Proëm.* iv. |
| Bégin, Mr. of *Metz*, General Principles of Pathological Physiology, | 237, 336, 428, 510 |

Page.

BOURDON, Mr. of *Paris*, on some Points in the Mechanism of Respiration and the Circulation of the Blood . . . . *Proëm.* xvi.
BREMSER, Dr. of *Berlin*, Treatise on Intestinal Worms . . . 71
BRESCHET, Dr. of *Paris*, account of a case of precocious Puberty . 346
BROOKE, Dr. W. of *Dublin*, on Cough and other Affections of the Lungs dependant on Disease of the Liver . . . . . 494
CARMICHAEL, Mr. R. of *Dublin*, case of Amputation at the Hip-joint . 488
Cases of Tracheotomy . . . . . . . . 489
CARSON, Dr. of *Liverpool*, on the Circulation of the Blood . *Proëm.* ix.
COLLIER, Mr. of *London*, notice of his Translation of the Pharmacopœia 433
CHAPMAN, Professor, of *Philadelphia*, on the Pathology and Treatment of Croup . . . . . . . . . 374
CHARPENTIER, Dr. of *Nevers*, account of a supposed case of spontaneous Combustion of the Human Body . . . . . . 347
COOKE, Dr. of *London*, History and Method of Cure of various species of Palsy . . . . . . . . . . 300
COPLAND, Dr. of *Walworth*, History of a case of Human Rumination, with an Inquiry into the Nature of the Process and some Phenomena of Digestion . . . . . . . . . 362
On the Yellow Fever . . . . . *Proëm.* xli.
COZE, Dr. of *St. Petersburg*, account of some Tumors of Nerves — lxxii.
DAVY, Sir H. on the Magnetic Effects produced by Electricity . . 83
DAVY, Dr. on the Urinary Secretion of some species of Rana . . 251
DAVY, Mr. E. Proposal of the Sulphate of Platinum as a Test for Gelatine 174
DEVEZE, Dr. of *Paris*, on the Yellow Fever . . *Proëm.* xxxvii.
DUNGLISSON, Dr. on some Phenomena of Vision . . — xxiii.
DUPUY, Prof. of *Alfort*, on the Inoculation of the Scab in Sheep . . 173
DUPUYTREN, Dr. of *Paris*, on the Re-union of Fractured Bones *Proëm.* lvii.
FALLOON, Dr. of *Dublin*, case of Diseased Heart in a Patient who had suffered Rheumatism . . . . . . . 497
FAULKNER, Sir A. BROOKE, Evidence given before a Committee of the House of Commons respecting the Contagion of the Plague 287, 467
FOHMANN, Dr. of *Heidelberg*, account of his Discoveries respecting the Lymphatic Vessels . . . . . *Proëm.* v.
FORBES, Dr. of *Penzance*, Comparative View of Mean Temperature at different Places in Great Britain . . . . . . 86
FOUQUIER, Dr. of *Paris*, on the Medicinal Qualities of the Acetate of Lead 252
FRANK, Dr. L. of *Vienna*, on the Plague of Egypt . *Proëm.* xlix.
FREER, Mr. of *Birmingham*, Histories of two cases of the Formation of Artificial Anus . . . . . . . . . 9
GILLESPIE, Dr. LEONARD, some observations on the Nature and Treatment of Venereal Diseases . . . . . 192
GIRARDIN, Dr. of *Paris*, on the Yellow Fever . . *Proëm.* xxxvii.
GLADSTONE, Dr. WM. Evidence given before a Committee of the House of Commons, respecting the Contagion of the Plague . . 35
GOOCH, Dr. of *London*, on the spontaneous Evolution of the Fetus. *Pro.* lxxxviii.
GRANVILLE, Dr. of *London*, Treatise on the internal Use of the Prussic Acid . . . . . . . . . . 45
Evidence given before a Committee of the House of Commons, respecting the Contagion of the Plague . . . . . 211
GRATTAN, Dr. of *Dublin*, on Medical Education, &c. . . . . 177
Case of Gangrene occasioned by the use of Mercury . . 492
GUANI, Dr. on the new Italian Doctrine . . . . *Proëm.* li.
HADEN, Mr. of *Sloane-street*, observations on the Medicinal Qualities of the Colchicum Autumnale . . . . . . 218
HARLESS, Prof. of *Bonn*, on the recent proferred Improvements in Medicine *Proëm.* viii.
HARRISON, Dr. EDWARD, of *London*, observations respecting the Nature and Origin of the common species of Disorder of the Spine; with Critical Remarks on the Opinions of former Writers on this Disease . . . . . . . . . . 103

Page.

HARRISON, Dr. W. of *Dublin*, case of Hemorrhage, supposed to originate from the Spleen . . . . . . . . . 473

HARRISON, Mr. JOHN, of *London*, an account, with Engravings, of some forms of Venereal Disease . . . . *Proëm.* lxi.

HERVEZ DE CHEGOIN, Dr. account of a peculiar species of Fracture of the Neck of the Femur . . . . . . — lxx.

HOME, Sir EVERARD, of *London*, on the Structure of the Skin of the Negro 83

HORNER, Dr. of *Philadelphia*, History of a case of Lumbar Abscess, attended with preternatural Anus in the Groin . . . 458

HOWELL, Mr. of *Wimbledon*, account of a rare case of Mal-formation in a Child . . . . . . . . . . . 199

HUTCHISON, Mr. A. COPLAND, of *London*, on the Treatment of Ulcers of the Legs by Strapping . . . . . . . 32

IRELAND, Dr. of *Dublin*, case of Idiopathic Emphysema . . . 476

IRVINE, Dr. on the Yellow Fever . . . . *Proëm.* xxxvii.

JACKSON, Mr. of *Bolton-le-Moors*, description of a Bicephalous Fetus . 128

JACOBSEN, Dr. account of a new-discovered Nerve in the Ear *Proëm.* v.

JEMINA, Dr. of *Mondovi*, case of Angina Pectoris . . . — lvi.

JENNER, Dr. of *Berkley*, on Vaccine Inoculation . . . . . 277

JUKES, Mr. of *London*, account of some Osseous Tumors found in the Uterus of a Woman . . . . . . . 353

KINGLAKE, Dr. of *Taunton*, on Vaccine Inoculation . . . 265

On the Position of Fractured Limbs . . . . . 356

On Animal Excitability . . . . . . . 441

KLEIN, Dr. of *Stuttgardt*, case of Removal of the Thyroid Gland . 173

LASSAIGNE, Mr. of *Paris*, Analysis of the Asarum Europeum *Proëm.* lxxxix.

LALLEMAND, Dr. of *Montpellier*, on pultaceous Disorganization of the Brain . . . . . . . — lvi.

LE FORT, Dr. on the Yellow Fever . . . . — xxxvii.

LEGOUAIS, Dr. of *Paris*, on the Puerperal Fever . — lii.

LEGONPIL, Dr. of *Valognes*, an account of the Expulsion of a Portion of the Intestines . . . . . . — lxix.

LENDRICK, Dr. of *Dublin*, case of Recovery from the Effects of Corrosive Sublimate . . . . . . . . . 498

MAGENDIE, Dr. of *Paris*, several Memoirs on subjects of Physiology 255, 434, 520

MARLEY, Mr. MILES, of *London*, case of re-Union of two Portions of the Finger, after they had been for a considerable time separated . 134

On the use of Cubebs in Gonorrhœa . . . . . 463

M'KEEVER, Dr. of *Dublin*, case of Rupture of the Vagina, and Sloughing away of a portion of the Intestines, consequent on Parturition . 496

MECKELL, Prof. A. of *Halle*, on the Villosities of the Intestines *Proëm.* iii.

MINES, Mr. of *Diss*, case of Hydrophobia . . . . . . 280

MOREAU DE JONNES, le Chevalier, of *Paris*, on the Yellow Fever *Pro.* xxxvii.

MORTON, Earl of, account of a curious Hybrid from a Mare and a Quagga 172

MOTT, Dr. of *New-York*, further Remarks on his Case of Ligature of the Arteria Innominata . . . . . . . . 169

Account of two cases of Disunited Fractures successfully treated by Seton . . . . . . . . . . . ib.

NACQUART, Dr. of *Paris*, account of a case of Tran-position of the Thoracic and Abdominal Viscera . . . *Proëm.* lxxvi.

NICHOLL, Dr. WHITLOCK, of *Ludlow*, General Elements of Pathology *Proëm.* xxix. 225

On Affections of the Cranial Brain of Infants . . . 476

Account of a case of Melœna, cured by Spirit of Turpentine . 496

ORFILA, Dr. of *Paris*, proposal of a new Means of Discovery of some Mineral Poisons . . . . . . . . 200

PARISET, Dr. of *Paris*, on the Yellow Fever . . *Proëm.* lxxix.

PINEL, Dr. of *Paris*, Histories of cases of Inflammation of the Spinal Marrow . . . . . . . . . . 282

PRICHARD, Dr. of *Bristol*, on the connexion of Inflammation with Fever *Pr.* xlvii.

PRING, Mr. of *Bath*, History of a case of the successful Formation of an artificial Anus in an Adult . . . . . . 1

Page.
PROUT, Dr. of *London*, on the Nature and Treatment of Urinary Calculi 385
RAMSBOTHAM, Dr. of *London*, Observations in Midwifery . . 499
RATIER, Dr. of *Paris*, on the Buffy Coat on the Blood . . . 85
RAYER, Dr. of *Paris*, on the Coryza of Infants . . *Proëm.* lxxvii.
REESE, Dr. of *Baltimore*, on the Yellow Fever . . — xxxvii.
REVERE, Dr. on the Yellow Fever . . . . . — ib.
RITMEISTER, Dr. of *Powlowsk*, on the Febrifuge Qualities of the Lepidium Rudcrale . . . . . . . — xci.
ROBIQUET, Mr. of *Paris*, account of a new Preparation of Opium . 350
ROBINSON, Dr. of *Dublin*, cases of Varioloid Disease . . . 475
ROGET, Dr. of *London*, on some Phenomena of Vision . *Proëm.* xxvi.
ROSTAN, Dr. of *Paris*, on pultaceous Disorganization of the Brain — lii.
ST. VINCENT, Dr. of *Brest*, cases of Rupture of the Heart . . — lxxiii.
SANDWITH, Mr. of *Bridlington*, on Epidemic Fever . . — xlvii.
SHERWEN, Dr. of *Bath*, on the means of reducing Strangulated Hernia 19
SMERDON, Mr. of *Clifton*, on the Efficacy of Pressure in the cure of local Affections of the Leg . . . . . . 450
On the Position for Fractured Limbs . . . . 22
SMITH, Mr. of *Bristol*, on the relative Prevalence of Urinary Calculi in different Parts of Great Britain . . . *Proëm.* lxxxi.
STRAUB, Mr. of *Hofwyl*, on the sources of Iodine . . — lxxxix.
STEVENS, Dr. of *New York*, accounts of several Cases in Surgery 165—167
SWAN, Mr. of *Lincoln*, on the Treatment of morbid local Affections of Nerves 315
TARBES, Mr. account of a case of Human Rumination . *Proëm.* xxvii.
THACKRAH, Mr. of *Leeds*, History of a case of Hernia Cerebri . . 195
THOMSON, Mr. TODD, of *Sloane-street*, on the Preparation of the Colchicum Autumnale . . . . . . . . *Proëm.* xci.
TIEDMANN, Prof. of *Heidelberg*, account of a case of Duplication of several Muscles . . . . . . . . . — lxxvi.
TRAVERS, Mr. of *London*, Synopsis of the Diseases of the Eye . — lxvi.
TRIPE, Mr. of *Plymouth Dock*, Remarks on Mr. Smith's Statistic Inquiry respecting the frequency of Stone in the Bladder in Great Britain 275
VENTURI, Dr. some Pathological Observations . . . . 252
VETCH, Dr. of *London*, Treatise on Diseases of the Eye . *Proëm.* lxi. 135
VOGEL, Mr. of *Munich*, account of an Analysis of Human Bile containing Cholesterine . . . . . . . — lxxxviii.
WADE, Mr. of *London*, on the Position of Patients after the Operation for Hernia . . . . . . . . . . 456
WALKER, Mr. of *Oxford*, account of two Operations for Aneurism . . 254
WALLIS, Mr. of *Langport*, some observations respecting Injuries of the Brain 208
WALTHER, Mr. of *Bonn*, account of some Lacrymal Calculi *Proëm.* lxxvii.
WATTS, Dr. of *New York*, on the Yellow Fever . . . — xxxvii.
WHITE, Mr. JAMES, of *Bath*, on the Structure and Use of the Thyroid Gland in the Horse, &c. . . . . . . . . 284
Observations on the Structure and Functions of the Udder of the Cow 462
WILSON, Mr. JAMES, of *London*, on the Vascularity of the Teeth. *Pro.* vii. xxviii.
On Fracture of the Neck of the Femur . . — lxxv.
YOUNG, Mr. SAMUEL, of *London*, on the Effects of Pressure in Cancer and some other Diseases . . . . . . 27, 189
ZECHINELLI, Dr. of *Bulluno*, on a certain species of Venereal Disease *Proëm.* lx.

THE LONDON

### Medical and Physical Journal.

1 OF VOL. XLV.] JANUARY, 1821. [NO. 263.

### NOTICE.

*The next of the series of* PRŎEMIA *to the several volumes of this Journal, (which commenced with that to the forty-third volume,) comprising a History of the Progress of Medicine and its auxiliary Sciences for the half-year immediately previous to the period of their production, respectively, will be published on the last day of January. One of the especial intentions of those* Prŏemia, *is to present a comprehensive view of the state and progress of Medicine throughout Europe generally, and in the United States of America; an object that cannot be effected in the regular monthly Numbers of the Journal, because of the small extent of space which can be there appropriated to this purpose.*

